# Causes of Delayed Transfers From the Emergency Department to Speciality Units: A Prospective Observational Study at a Tertiary Care Centre

**DOI:** 10.7759/cureus.108990

**Published:** 2026-05-16

**Authors:** Pooja Thaware, Anshu P Lakra, Ankita, Anirudh Elayat, Saurabh Trivedi, Jitendra Kushwaha

**Affiliations:** 1 Trauma and Emergency Medicine, All India Institute of Medical Sciences, Bhopal, Bhopal, IND; 2 Anesthesiology and Critical Care, All India Institute of Medical Sciences, Mangalagiri, Mangalagiri, IND

**Keywords:** emergency service, hospital, hospital crowding, patient admission, patient transfer, tertiary care centres

## Abstract

Background: Delays in transferring patients from the emergency department (ED) to speciality units cause overcrowding and poor outcomes. The causes of prolonged ED boarding exceeding 24 hours remain understudied, especially in Indian tertiary centres.

Methods: This prospective observational study over six months at a central India tertiary care hospital enrolled adults (≥18 years) staying ≥24 hours in the ED without speciality transfer (n=510). A delay was defined as time from ED registration to the speciality unit's documented transfer decision exceeding 24 hours. Causes were classified as patient-related (e.g., poor prognosis: Sequential Organ Failure Assessment (SOFA) ≥ 13, cancer patients) or system-related (e.g., ICU/high-dependency unit (HDU) bed waits, interdepartmental disputes). We assessed ED stay, hospital length of stay (LOS), and discharge status. Data were analysed using descriptive statistics.

Results: System causes predominated (70%): speciality ICU/HDU bed waits in 169 (33.1%) patients and interdepartmental disputes in 166 (32.5%). Poor prognosis ranked third in 135 (26.5%) patients. Of 510 patients boarded >24 hours, 438 (85.9%) received delayed transfer (delayed transfer group) and 72 (14.1%) stayed in ED without transfer (non-transferred group). Among the 438 transferred patients, median ED stay before transfer was 51 hours (IQR 48-72 hours); 310 (70.7%) were transferred within 72 hours. In the delayed group, 301 (68.7%) were discharged, 71 (16.2%) died, and 66 (15.1%) left against medical advice; median hospital LOS was seven days. The non-transferred group had a shorter LOS (median 2.5 days).

Conclusion: Despite efficient 24-hour unit rotations, pre-decision system bottlenecks drive prolonged ED boarding. Hospital-wide bed dashboards, dedicated surge ICU beds, and ED transfer authority for long boarders would improve patient flow in resource-constrained settings.

## Introduction

Timely transfer of patients from the emergency department (ED) to appropriate speciality units is essential for continuity of care, optimal resource use, and better clinical outcomes. Delayed transfers leave patients boarded in the ED after acute stabilization, which harms individual care and reduces overall emergency service efficiency.

ED overcrowding is a global issue driven by multiple linked factors [1,2]. These include rising patient volumes, poor primary care access, delays in tests and consultations, staff and bed shortages, and flaws in discharge and admission processes [3]. Frameworks worldwide group these issues into input, throughput, and output domains. Proposed solutions include quick triage, observation units, better communication, and hospital-wide bed management [4]. Delays have been consistently associated with longer hospital stays, higher morbidity, and increased mortality

Prior studies have examined delays in securing ED beds and their ties to worse outcomes, as well as reasons for late ICU transfers after admission decisions [4-6]. However, little research covers the causes and effects of prolonged ED boarding and patients staying over 24 hours without transfer to a suitable speciality unit. These patients get neither definitive speciality care nor discharge, which sustains ED overcrowding and highlights systemic problems. This study aimed to prospectively identify and categorise causes of ED boarding exceeding 24 hours in an Indian tertiary centre, where such data remain scarce.

## Materials and methods

Study design

This was a single-centre, prospective, observational study conducted over six months at a tertiary care teaching hospital in central India. The emergency department has 31 beds, including 10 dedicated pediatric beds, and manages an average daily patient load of approximately 193 visits. The study was approved by the Institutional Human Ethics Committee (LOP/2024/P24/006). The study protocol was registered prospectively with the Clinical Trials Registry-India (CTRI/2024/03/064319). Written informed consent was obtained from all participants or their legally authorised representatives. The study was conducted in accordance with the ethical principles outlined in the Declaration of Helsinki.

Speciality units

Speciality units included all MD/MS and DM/MCh departments at the institute.

Participants

All patients aged ≥18 years who remained in the ED for ≥24 hours without documented transfer to a specialty unit during the six-month study period were consecutively enrolled. The final sample of 510 represents a total enumeration of eligible patients over this period. We excluded patients <18 years and pregnant women. Patients aged <18 years and pregnant women were excluded because delayed transfer in these groups was not identified as a significant problem in our emergency department setting. A delay was defined as time from ED registration to the speciality unit's documented transfer decision exceeding 24 hours.

Aim and objectives

The aim was to document causes of delayed patient transfers from the ED to speciality units. The primary objective was to identify causes of delays exceeding 24 hours. Secondary objectives were to measure ED stay duration before transfer, total hospital length of stay (LOS), and patient outcomes during hospitalisation.

Adult patients aged ≥18 years of either gender who remained boarded in the emergency department for more than 24 hours since the time of registration were included. Patients aged less than 18 years and pregnant patients were excluded from the study.

Data collection

**Figure 1:**
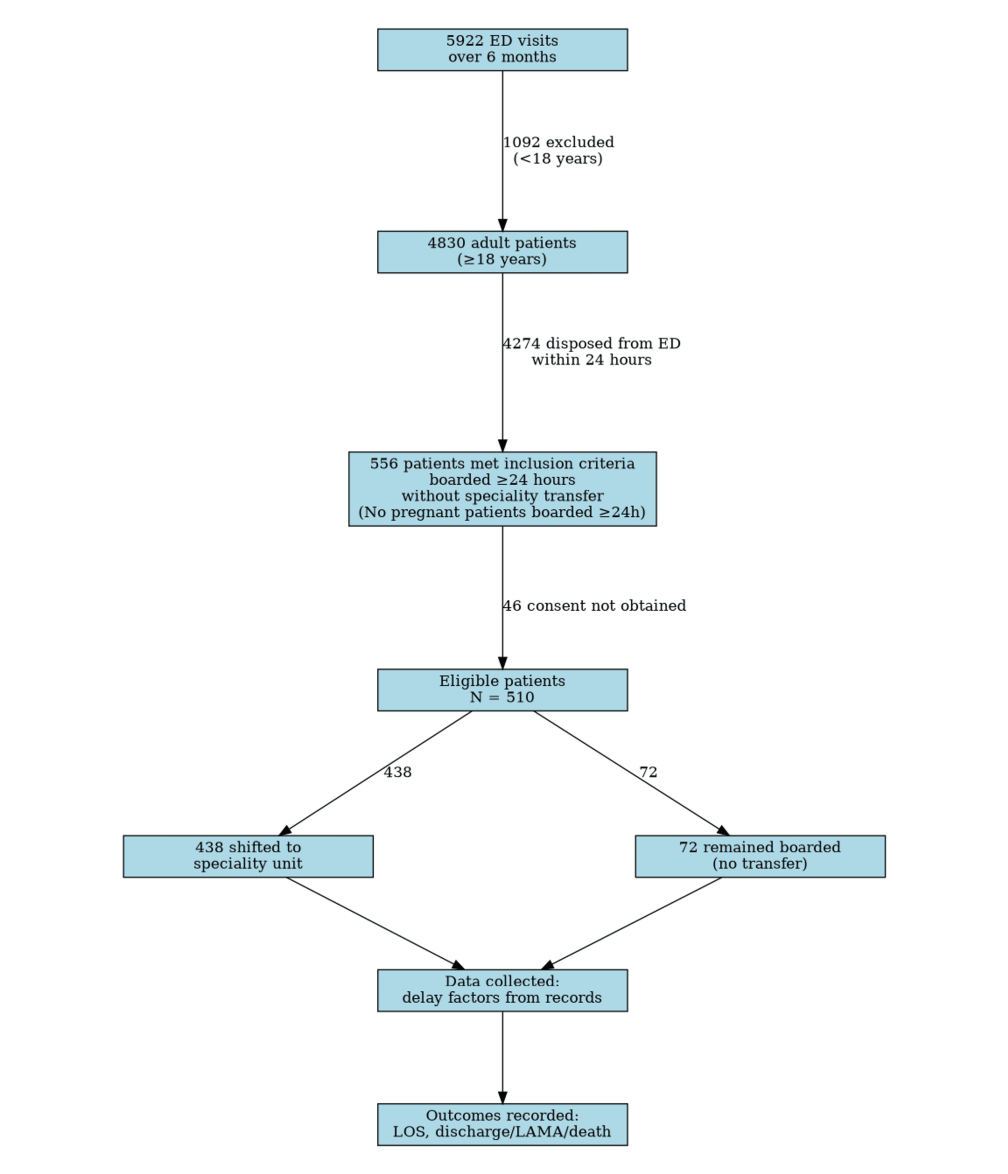
Cohort flowchart showing patient selection process. LOS: length of stay, LAMA: left against medical advice

Data were prospectively collected from patient medical records using a structured proforma (Figure 1).

Causes of delay fell into two main categories. Each patient was assessed for all applicable causes, and more than one cause could be assigned to a single patient where multiple contributing factors were identified. Frequencies are therefore reported as proportions of the total cohort (n=510) and may cumulatively exceed 100%.

Category A: Patient-Related Causes

Patients with poor prognosis: Critically unstable patients with multiple organ dysfunction syndrome (MODS) on high inotropic or vasopressor support (Sequential Organ Failure Assessment (SOFA) ≥13) showing severe physiological decline and high mortality risk with low recovery odds (Table 1).

**Table 1:** Sequential Organ Failure Assessment Score MAP: mean arterial pressure, UO: urine output, PaO_2_: partial pressure of oxygen, FiO_2_: fraction of inspired oxygen

Organ system	Score 0	Score 1	Score 2	Score 3	Score 4
Respiration (PaO₂/FiO₂, mmHg)	>400	≤400	≤300	≤200 (with respiratory support)	≤100
Coagulation (Platelets, ×10^3^/mm^3^)	>150	≤150	≤100	≤50	≤20
Liver (Bilirubin, mg/dL)	<1.2	1.2–1.9	2.0–5.9	6.0–11.9	≥12.0
Cardiovascular (MAP, mmHg & adrenergic agents administered for at least one hour, mcg/kg/min)	No hypotension	MAP <70	Dopamine ≤5 or Dobutamine (any dose)	Dopamine >5 or epinephrine ≤0.1 or norepinephrine ≤0.1	Dopamine >15 or epinephrine >0.1 or norepinephrine >0.1
CNS (Glasgow Coma Scale)	15	13–14	10–12	6–9	<6
Renal (Creatinine, mg/dL or UO)	<1.2	1.2–1.9	2.0–3.4	3.5–4.9 or UO <500ml/d	≥5.0 or UO <200ml/d

Patients with poor prognosis were assessed using the SOFA score (Vincent et al., 1996), with neurological status evaluated using the Glasgow Coma Scale (GCS) (Teasdale and Jennett, 1974) [7,8].

Carcinoma patients with no active disease-directed intervention planned: Metastatic or non-metastatic cases where no curative treatment was planned during the ED visit; limited to acute symptom management.

Long-term bedridden patients with acute symptoms: Chronically bedridden patients presenting with new acute issues, such as fever, breathlessness, or altered sensorium etc.

Surgical speciality patients requiring conservative management: Surgical cases needing medical management in the ED, without immediate surgery.

Step-down care transfers: Patients from other facilities ICUs or HDUs needing lower-level post-acute care.

Category B: System-Related Causes

Financially exhausted private-to-public transfers: Patients transferred from private facilities unable to afford further care but still needing acute treatment.

Patients awaiting ICU/HDU bed under designated speciality: Patients assigned to a speciality but waiting for intensive bed availability.

Interdepartmental/multi-departmental dispute/denial cases (including polytrauma): Cases where two or more departments disputed primary responsibility, needing coordination until the lead department accepted.

Sample size

Consecutive sampling was used, and all eligible patients presenting during the six-month study period who fulfilled the inclusion and exclusion criteria were enrolled consecutively. Consecutive sampling was used to ensure inclusion of all eligible patients presenting during the study period, thereby reflecting real-world emergency department boarding patterns in routine clinical practice.

Statistical analysis

All statistical analyses were performed using IBM SPSS Statistics, Version 30.0 (IBM Corp., Armonk, NY, USA). Continuous variables are presented as mean ± standard deviation (SD) for normally distributed data and as median with interquartile range (IQR) for skewed data. Categorical variables are expressed as frequencies and percentages. 

## Results

Patient characteristics and causes of delayed transfer

Of 5,922 ED admissions over six months, 510 patients (8.6%) had delayed transfers to speciality units. Mean age was 49.9 years (SD 12.3). Males outnumbered females (308 [60.4%] vs 202 [39.6%]).

System-related causes were most common. Patients awaiting ICU/HDU beds in assigned speciality units (169 [33.1%]) and interdepartmental disputes or denials (including polytrauma; 166 [32.5%]) accounted for nearly two-thirds of delays. Poor prognosis was the top patient-related cause (135, 26.5%) (Table 2). 

**Table 2:** Causes of delayed ED transfer (n=510).

Category	Cause	Total (n=510), n (%)
B (System-related)	Awaiting ICU/HDU bed	169 (33.1)
B (System-related)	Interdepartmental dispute/denial	166 (32.5)
A (Patient-related)	Poor overall prognosis	135 (26.5)
A (Patient-related)	Carcinoma, no active intervention	47 (9.2)
A (Patient-related)	Step-down from other facility	30 (5.9)
B (System-related)	Financially exhausted private-to-public	25 (4.9)
A (Patient-related)	Surgical, conservative management	20 (3.9)
A (Patient-related)	Long-term bedridden with acute symptoms	8 (1.6)

Of 510 patients boarded >24 hours, 438 (85.9%) eventually transferred (delayed transfer group), and 72 (14.1%) did not (non-transferred group).

Emergency department stay

In the delayed transfer group (n=438), median ED stay before transfer was 51 hours (IQR 48-72 hours). Of these, 310 (70.7%) transferred within 72 hours; 30 (6.8%) stayed ≥96 hours (Table 3).

**Table 3:** Total time in ED before transfer to speciality unit (n=438)

Time duration in hours	Number of patients, n (%)
24–<48	171 (39.0)
48–<72	139 (31.7)
72–<96	98 (22.4)
≥96	30 (6.8)

Hospital length of stay

Delayed transfer patients (n=438, 85.9%) had mean hospital LOS of 10.5 days (SD 9.7); 231 (52.7%) stayed seven or less days, and 42 (9.6%) stayed >21 days. Non-transferred patients (n=72, 14.1%) had shorter median LOS of 2.5 days; 68 (94.4%) stayed seven or less days (Table 4).

**Table 4:** Hospital length of stay (LOS) by transfer group.

Length of stay	Number of patients n (%) among patients with delayed transfer (n=438)	Number of patients n (%) among patients with non-transfer (n=72)
LOS ≤7 days	231 (52.7)	68 (94.4)
LOS 8-14 days	105 (24.0)	3 (4.2)
LOS 15-21 days	60 (13.7)	1 (1.4)
LOS >21 days	42 (9.6)	0

Patient outcomes

Among delayed transfer patients, 301 (68.7%) were discharged from speciality units, 71 (16.2%) died, and 66 (15.1%) left against medical advice (LAMA). For non-transferred patients, 47 (65.3%) were discharged directly from ED, 13 (18.1%) died, and 12 (16.7%) LAMA (Table 5).

**Table 5:** Patient outcomes LAMA: left against medical advice

Outcome	Number of patients n (%) among patients with delayed transfer (n=438)	Number of patients n (%) among patients with non-transfer (n=72)
Discharge	301 (68.7)	47 (65.3)
Death	71 (16.2)	13 (18.1)
LAMA	66 (15.1)	12 (16.7)

Descriptive comparison showed patients who died or left against medical advice had longer median ED boarding times than those discharged (72 hours vs 48 hours); however formal statistical comparison was beyond the scope of this study's objectives.

## Discussion

This prospective observational study at a tertiary care centre in central India found that system-related factors accounted for most delayed transfers from the emergency department to speciality units beyond 24 hours. The most common causes were awaiting speciality ICU/HDU beds and interdepartmental disputes or denial cases, while poor prognosis was the leading patient-related factor. Most transferred patients experienced prolonged ED boarding despite dedicated 24-hour speciality unit rotations, highlighting that pre-transfer system bottlenecks were the major contributors to delayed patient flow

Emergency department overcrowding refers to a situation where the demand for emergency services exceeds the available ED treatment capacity, including beds, staffing, and resources. At our institute, the ED has 31 beds, including 10 dedicated pediatric beds, and manages an average of approximately 193 patient visits per day.

System delays aligned with global models of ED overcrowding, which use input-throughput-output frameworks where output blocks like bed shortages and transfer delays dominate. Savioli et al. highlighted inpatient bed shortages as the main issue, favouring hospital-wide bed management over ED expansion [3]. Lindner and Woitok noted output bottlenecks (70-80% of crowding) from inpatient bed shortages, prolonged boarding, discharge delays (e.g., late rounds, medication reconciliation, transport), and weekend gaps [4]. They measured boarding as hours from admission decision to ward transfer, exceeding throughput issues like triage or input surges. Their review promoted macro-strategies (hospital-wide bed management, reverse triage, full-capacity protocols reducing waits 40-50%) over ED fixes mirroring our ICU/HDU waits and interdepartmental disputes, even with efficient 24-hour unit rotations.

Bosco et al., in India, found delays in 67.3% of ED-to-ICU transfers >30 minutes post-decision, mainly ED factors (54% e.g., staff/trolley/equipment shortages), then patient (20%; e.g., consent/finance/tests), administrative (14%; e.g., medicolegal/scheme clearance), and ICU (12%; e.g., bed/ventilator prep) [5]. Regular feedback reduced transfer delays substantially without adversely affecting mortality outcomes.

Unlike Bosco et al.'s post-decision focus, our study measured delays from ED registration to speciality transfer decision exceeding 24 hours. Unit rotations cleared most cases efficiently; none of the similar factors (e.g., test delays, staff shortages, clearances) caused >24-hour delays in our setting. This underscores our novel view of pre-decision bottlenecks like ICU/HDU waits and disputes, suggesting feedback and policy could improve both phases for better flow.

Interdepartmental disputes/unclaimed cases echoed Abraham et al.'s qualitative findings on ED to MICU barriers in the pre-transfer phase [6]. ED teams faced repeated rejections from MICU due to bed shortages, slow decisions, and incomplete data (e.g., missing vitals/labs), stemming from poor handoffs of responsibility and control. These match our ICU/HDU waits and could improve via checklists, videophones, "crash beds," and standard protocols.

Our most common cause ICU/HDU bed waits in 169 (33.1%) patients aligns with Peltonen et al.'s cluster of transport and bed issues, staffing shortages, and patient-related problems identified from 333 delay phrases [9]. Our poor prognosis cases (SOFA ≥13) (Table 5) matched their health deterioration group. Their semi-supervised analysis covered 86% of phrases but lacked stay durations; our prospective data linked these to >24-hour boarding.

Patient factors like poor prognosis matched Venkatesh et al., who linked uninsured/complex cases to higher delay odds from access gaps [10].

Jones et al. analysed more than five million ED visits over two years, finding delays from arrival to bed decision raised 30-day mortality linearly from five hours (8% higher risk at six to eight hours; one extra death per 82 delayed patients) [11]. We measured boarding from registration to transfer decision >24 hours.

Strengths and limitations

Prospective design with structured proforma reduced recall bias, capturing real-time causes in a high-volume ED with unique Indian unit rotations ensuring most <24-hour claims. Patient (A) vs system (B) categories gave clear prevalence for policy. Limitations were that a single-centre design limits generalizability and multicentre studies are needed; unblinded data collection risks observer bias; cause categorisation was performed by the research team without formal inter-rater reliability testing; and long-term patient outcomes beyond hospitalisation were not tracked. Another limitation was that risk ratio analysis was not performed, as the study objectives were primarily descriptive; further analytical studies may explore such associations in greater detail.

Clinical and policy implications

For patients awaiting specialty ICU/HDU beds, the Erlanger eSTAR model offers a practical solution: a dedicated surgical transfer unit bypassed ED for >1,400 non-trauma cases over 19 months, averting 1,250 ED visits and 850 boarder days via protocol training, surgeon education, and electronic health record flags [12]. This leadership approach sparked by boarder data review and a site visit shows process changes beat infrastructure needs.

Our institute could add a hospital-wide bed dashboard for ED access, with ICU surge beds for urgent needs. Hospital-wide bed dashboards would enhance real-time bed availability visibility, thereby expediting patient turnover across departments. Dedicated surge ICU beds would ensure requisite capacity for critically ill boarders without necessitating major infrastructural expansion.

Clear protocols (e.g., organ-specific routing) would cut disputes (31.5%). ED physicians' authority to transfer >24-hour boarders to the most appropriate speciality unit following consultation would ease overcrowding and flow in resource-limited settings.

## Conclusions

This prospective observational study found that system-related factors were the predominant causes of delayed transfer from the emergency department to speciality units beyond 24 hours. The most common causes were awaiting speciality ICU/HDU beds and interdepartmental disputes, while poor prognosis was the leading patient-related factor.

Despite dedicated 24-hour speciality unit rotations at our institute, prolonged ED boarding persisted primarily because of pre-transfer system bottlenecks rather than deficiencies in the transfer process itself. Most patients who experienced delayed transfer remained boarded in the ED for prolonged durations before speciality acceptance.

These findings highlight the importance of improving inpatient bed coordination and streamlining interdepartmental transfer processes to reduce prolonged ED boarding in tertiary care emergency settings.
